# A physicochemical model of X-ray induced photodynamic therapy (X-PDT) with an emphasis on tissue oxygen concentration and oxygenation

**DOI:** 10.1038/s41598-023-44734-y

**Published:** 2023-10-19

**Authors:** Farideh. S. Hosseini, Nadia Naghavi, Ameneh Sazgarnia

**Affiliations:** 1 Department of Electrical Engineering, Ferdowsi University of Mashhad, Mashhad, Iran; 2 Medical Physics Research Center, Mashhad University of Medical Sciences, Mashhad, Iran; 3 Department of Medical Physics, Faculty of Medicine, University of Medical Sciences, Mashhad, Iran

**Keywords:** Cancer, Computational biology and bioinformatics, Medical research, Engineering, Mathematics and computing, Nanoscience and technology

## Abstract

X-PDT is one of the novel cancer treatment approaches that uses high penetration X-ray radiation to activate photosensitizers (PSs) placed in deep seated tumors. After PS activation, some reactive oxygen species (ROS) like singlet oxygen (^1^O_2_) are produced that are very toxic for adjacent cells. Efficiency of X-PDT depends on ^1^O_2_ quantum yield as well as X-ray mortality rate. Despite many studies have been modeled X-PDT, little is known about the investigation of tissue oxygen content in treatment outcome. In the present study, we predicted X-PDT efficiency through a feedback of physiological parameters of tumor microenvironment includes tissue oxygen and oxygenation properties. The introduced physicochemical model of X-PDT estimates ^1^O_2_ production in a vascularized and non-vascularized tumor under different tissue oxygen levels to predict cell death probability in tumor and adjacent normal tissue. The results emphasized the importance of molecular oxygen and the presence of a vascular network in predicting X-PDT efficiency.

## Introduction

Cancer is the second cause of death in the world after cardiovascular disease^1^. Among the various methods proposed for cancer treatment, radiotherapy (RT) utilizes ionizing radiation to fatally damage tumor cells. High tissue penetration of X-rays induce DNA breaks, resulting efficient cancer cell destruction^2^. Simultaneously, ionizing radiation induce side effects for surrounding normal tissues^3^. So, it is a challenging problem to balance between inhibiting tumor growth and reducing side effects to normal tissue. To achieve this goal, the concept of therapeutic efficiency has been defined as the ratio of cell death in tumor to healthy tissue. There are different factors affect the efficiency of RT such as tissue morphological characteristics (includes tissue oxygenation, Cell division speed, …), nature of radiation (dose, linear energy transfer, …) and presence of substances that affect the cell response to radiation (like radiosesitizer and radioresistant agents)^4^. Among these, radiosensitizers are chemical agents increase lethal effects of radiation^5^ and significantly lowering the radiation exposure necessary for tumor regression^6^.

In contrast to RT, photodynamic therapy (PDT) is a relatively new cancer treatment approach utilizes intermediate energies of electromagnetic spectrum (optical radiation) to produce cytotoxic ROS^7^. PDT interactions need three components of oxygen, PS and light with an appropriate wavelength to damage cells^8^. In the absence of each component, PDT interactions will not initiate. Thus, PDT is recognized as a safe, low toxicity and highly selective method of cancer treatment^7^. However, PDT is restricted to accessible tissues due to limited penetration depth of light.

X-PDT, on the other hands, is a novel approach can be used for treatment of cancer^9^. X-PDT utilizes nanocomposites include some nanoscintillators to locally convert X-rays to optical luminescence and activate conjugated PSs. In presence of oxygen, the activated PS, generate ROS to damage cancer cells^8,10^. Therefore, there are two important factors that affect the efficiency of X-PDT: first, scintillator light yield transforming X-ray into light, and then efficiency of the emitted photons being absorbed by the PS.

Moreover, metallic nanoscintillators increase local dose by selectively scattering and absorbing X-rays causing localized damage to DNA^5,11^. As a result, X-PDT combines RT and PDT to improve therapeutic outcome and reduces radiation damage to normal tissues^12^. X-PDT attacks both DNA and membrane of cancerous cells causing more effective damage than conventional RT or PDT alone^13^. With the growth of nanotechnology, the idea of using nanoscintillators for activating adjacent PSs introduced by Chen et al. in 2006^14^. Since then further efforts have been made by different researchers as reviewed in^9^. However, there are still some challenges for the clinical use of X-PDT^9^ and mathematical modeling will be able to response some of them.

Morgan et al. in 2009^15^ made the first attempts to estimate the amount of produced ROS by X-PDT. Although many uncertainties existed in their simulations, it was a first attempt and raised valid questions about the practicality of X-PDT as an effective treatment option^9^.

Bulin et al. in 2015^16^ proposed an alternative model using GEANT4-based Monte Carlo simulations to numerically estimate the spatial energy distribution in a macroscopic volume of water loaded with nanoscintillators. The results showed at physiological NP concentrations, biological effects of radiation are primarily mediated by water, the most abundant molecule in biological tissues and absorption of energy by NPs is mainly driven by inelastic electron scattering and is nearly independent of the nanoparticle X-ray stopping power^9^. Following these results, Klein et al. in 2019^17^ proposed a simplified electron cross section model to describe luminescence yield of NPs in a mix environment including water and nanoscintillators. They estimated that luminescence yield of NPs can be approximated as a function of the radiation dose, NP concentration, scintillator light yield and the electron cross-sections for tissue and the NP material. This model provides an upper bound for the actual number of scintillation photons emitted and is a better predictor of X-PDT efficacy. However, electron cross-section model as well as before theoretical models of X-PDT are not able to fully explain the X-PDT efficacy reported during pre-clinical tests^9^. The current models most predict the number of ^1^O_2_ per cell over a wide excitation energy range, which is below the required 10^7^ ^1^O_2_ PDT threshold for cell killing. This is while experimental studies demonstrated that X-PDT is more than just a PDT derivative but it is essentially a PDT and RT combination^12^. Moreover, the current models of X-PDT most focused on the interaction of ionizing radiation with nanoscintillators and generated light with PSs to estimate ^1^O_2_ concentration and predict produced ^1^O_2_ based on some physical parameters such as radiation dosage, nanoscintillator concentration, scintillator light yield and tumor volume^15–18^. Indeed, none of them investigate effects of tumor microenvironment on the treatment efficiency while it is known that some chemical parameters like molecular oxygen has a significant impact on the success or failure of cancer treatments^19,20^.

**Figure 1:**
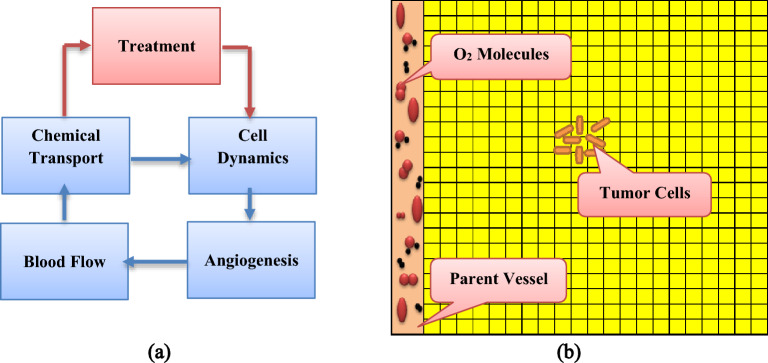
Overview of the model includes (**a**) Block diagram of the five compartment model and (**b**) Schematic of simulation space.

In the present study, we attempted to model cancerous cells treated with X-PDT through a feedback of tissue oxygen content. For this purpose, we first developed the mathematical model of tumor growth and then stablished a link between treatment parameters and physiological conditions of tumor environment. Due to the key role of molecular oxygen in RT effectiveness^21^ and ^1^O_2_ production in PDT^15^, we do take care of oxygen and its dynamics as the main nutrient in tumor environment. We have developed a physicochemical model of X-PDT to incorporate molecular oxygen as one of the main factors determining the amount of produced ROS and also effectiveness of X-PDT^18^. In this regards, we considered a five compartment model (Fig. 1) including chemical transport, cancer cells dynamics, angiogenesis, blood flow and treatment models.

## Results

**Figure 2:**
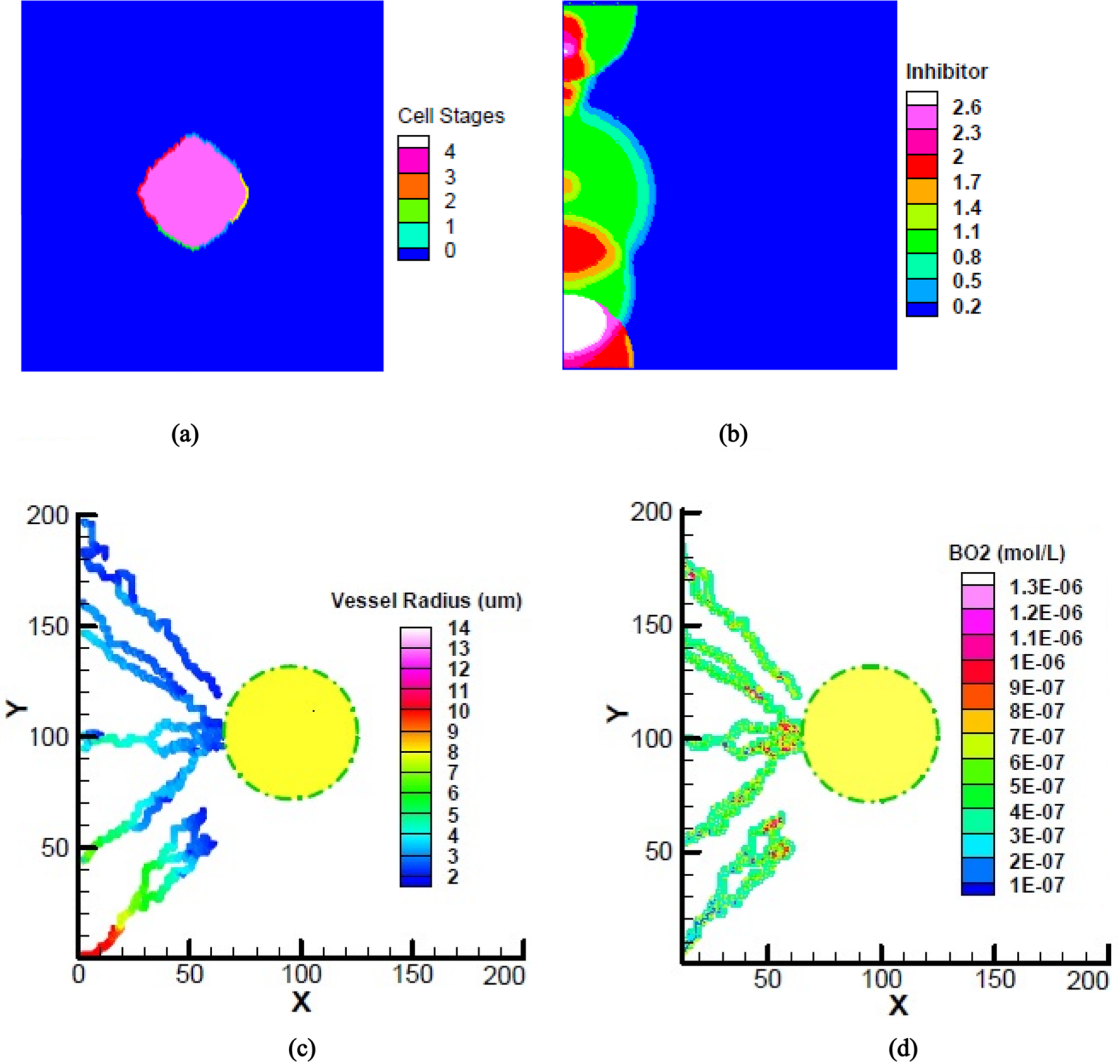
A snapshot of initial results of the coupled model includes: (**a**) tumor cell stages at the end of avascular growth phase (**b**) inhibitor distribution produced randomly around the parent vessel **(c)** vessel radius **(d)** Contribution of the produced capillary network in oxygen supply.

Figure 2 shows initial results of the coupled model after the growth of 9 initial tumor cells includes (a) the tumor stages at the end of avascular growth phase after 41.6 days (b) inhibitor distribution produced randomly around the parent vessel as well as sprouts initiated from the center of each semi-circle (c) distribution of vessel radiuses and (d) oxygen production rates by the new microvessel structure after 141.6 days. According to the Fig. 2(a), tumor includes 3221 cells that 2304 quiescent cells placed at the internal layers and 917 proliferative cells at the external layer. Production of VEGF by the hypoxic tumor cells initiates angiogenesis process with sprouting 8 sprouts along the parent vessel and production of inhibitor around each new sprout (Fig. 2b). The activated sprouts then move towards the VEGF source and form a new microvessel network to satisfy tumor cell demands. As shown in Fig. 2(c), the newly formed vessels have smaller diameters than the older ones (across x axis) and also vessel diameters vary depending on their positions along the parent vessel (across y axis) depending on the total applied stimulations S_total_. Different branching patterns among 8 activated sprouts are also related to the various amount of S_total_ applied to each sprout tip. Contribution of new vessels in providing molecular oxygen is shown in Fig. 2(d) and illustrates that lower radiuses of vessel segments as well as more segment connections provide more oxygen to the tumor.

**Figure 3:**
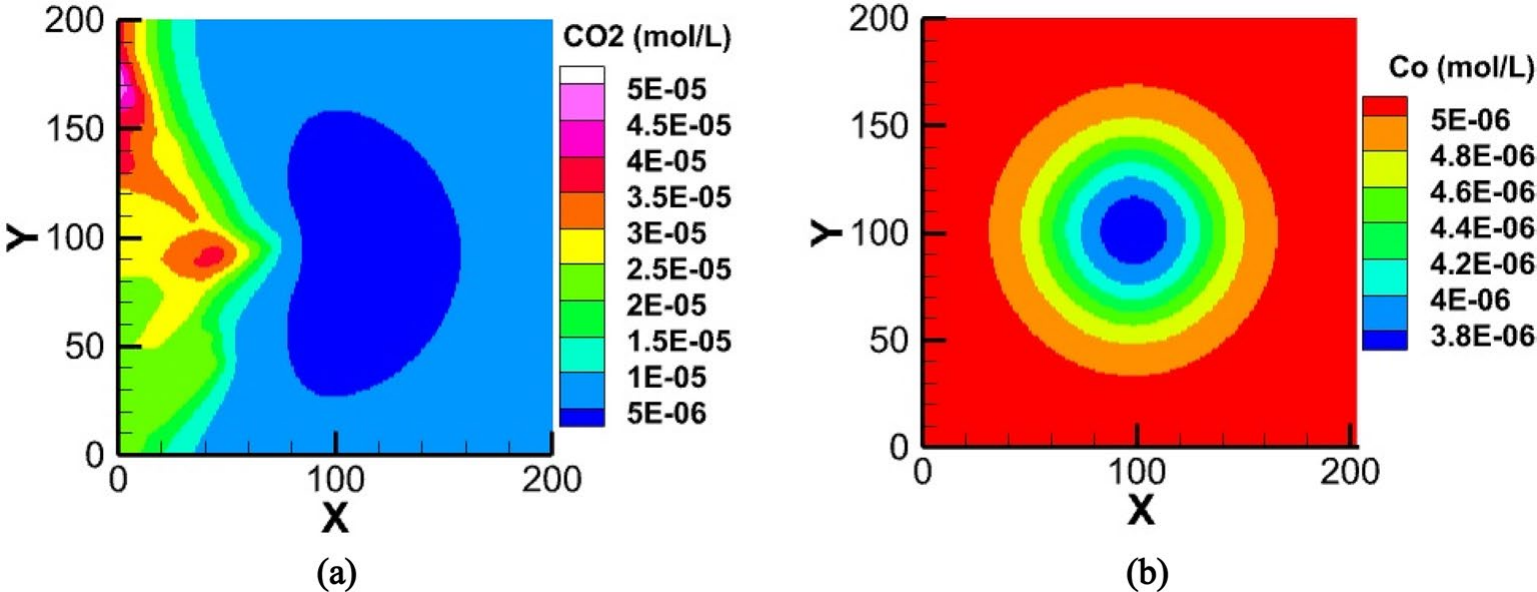
Distribution of oxygen in tumor and adjacent normal tissue, in a (**a**) vascularized tumor and (**b**) non-vascularized tumor.

Figure 3 shows distribution of oxygen in tumor microenvironment after 141.6 days. In a vascularized tumor of Fig. 3(a), a mass of oxygen and other nutrients transferred by the new vessels in response to the VEGF produced by hypoxic tumor cells. On the other hand, in a non-vascularized tumor of Fig. 3(b), that angiogenesis is not initiated (B_O2_ = 0), the lack of oxygen inside the tumor formed a layer of quiescent cells. The maximum level of oxygen in this situation corresponds to the minimum level of oxygen in the vascularized tumor of Fig. 3(a).

**Figure 4:**
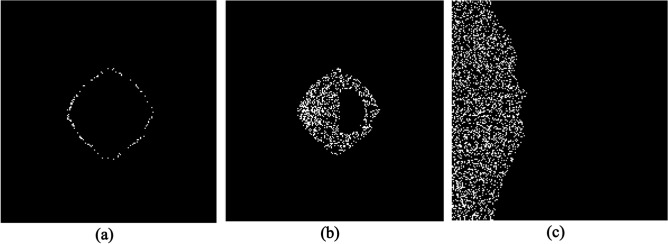
Proliferative (**a**) and quiescent (**b**) tumor cells as well as normal tissue (**c**) killed by ^1^O_2_ during X-PDT in a vascularized tumor. The white spots in (**a**) & (**b**) are tumor cells and in (**c**) are normal cells. The black spots are background area in all.

Now we investigate X-PDT efficiency for the two conditions of vascularized and non-vascularized tissue of Fig. 3. Figure 4 shows different cells of a vascularized tumor as well as normal tissue killed by ^1^O_2_ during X-PDT with dose 4 Gy and nanocomplex concentration of 4 mg/ml. We assume that half of drug concentration remains in the adjacent normal tissue. As illustrated, cells in regions with higher oxygen concentrations targeted more effectively by ^1^O_2_ both in tumor and normal tissue. According to the model considerations, new microvascular network initiated from the parent vessel on the left border of the domain, as a result cells in the left hand of the grid killed mostly by ^1^O_2_. Based on these results, in this vascularized tumor, about 22.73% of tumor cells and 8.39% % of normal cells damaged due to ^1^O_2_ toxicity. Among these, 2.54% of proliferative tumor cells (Fig. 4a) and 20.19% of quiescent tumor cells (Fig. 4b) have been damaged. Moreover, there is an isolated area inside the tumor where no cells have been killed and the treatment efficiency has been zero. Indeed, oxygen distribution determines the pattern of cell killing and hypoxia in this region acts like a defensive wall that protects tumor cells against ^1^O_2_ toxicity. This isolated area is not necessarily placed in the center of the tumor but is placed in the region with minimum oxygen distribution. As well, the lack of oxygen in a non-vascularized tumor does not provide necessary ^1^O_2_ threshold for cell killing. As a result, no tumor cells are destroyed by this X-PDT component in a non-vascularized tumor in the same condition.

**Figure 5:**
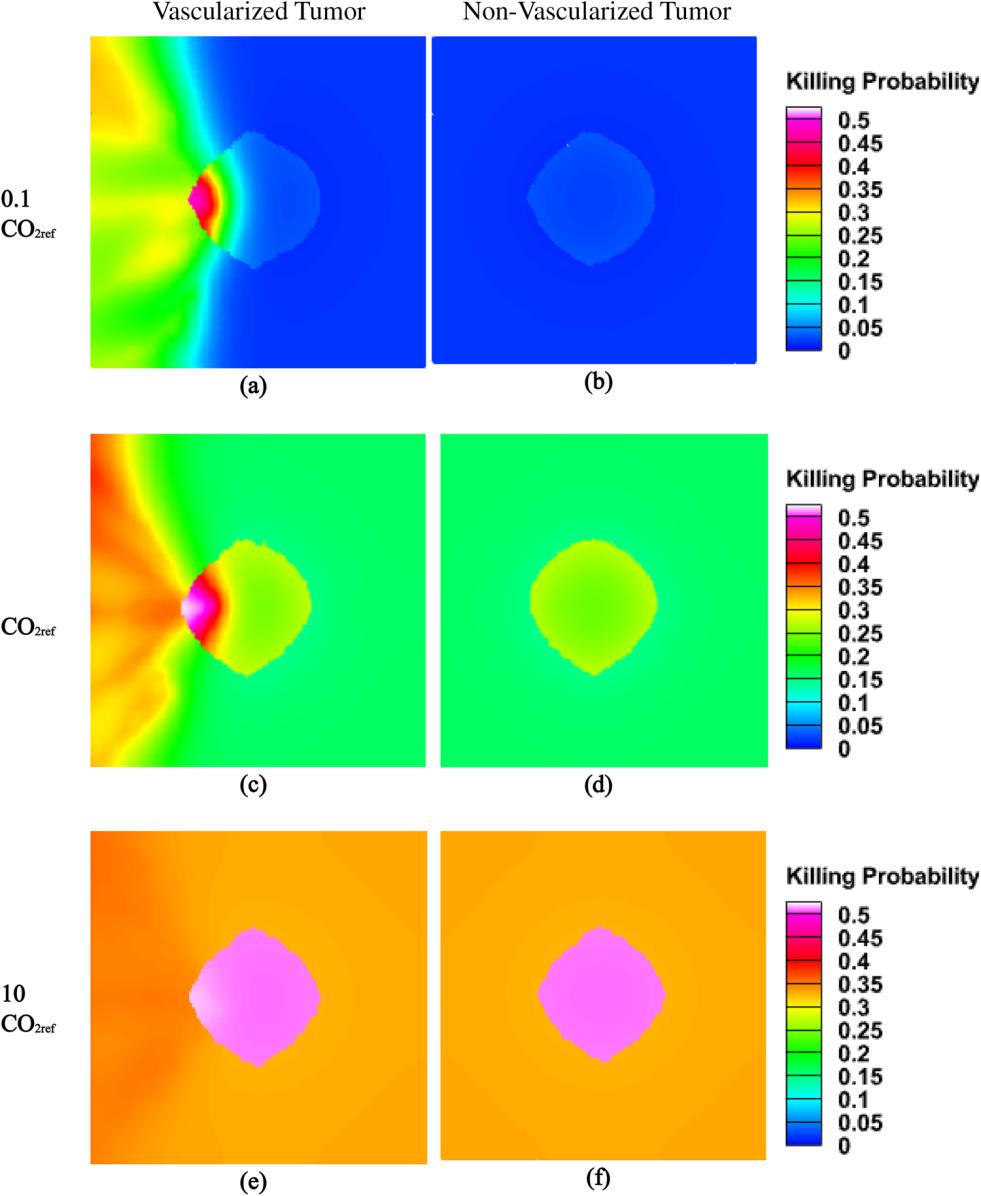
Probability of cell killing by ^1^O_2_ during X-PDT in a vascularized and non-vascularized tissue as well as different tissue oxygen levels under dose 4 Gy and nanocomplex concentration of 4 mg/ml.

Killing probability of ^1^O_2_ in a vascularized and non-vascularized tumor under different tissue oxygen concentrations investigated in Fig. 5. As shown, killing probabilities depend quantitatively and qualitatively on the tissue oxygen level as well as tissue oxygenation properties. With increasing background oxygen concentration, probability of cell killing also increases both in vascularized and non-vascularized tumor, until the level of oxygen concentration is too high (C_O2_ = 10 CO_2ref_) that there is no difference between a vascularized and non-vascularized tumor in killing probability.

Tissue oxygenation properties also determine killing probabilities for tumor and adjacent normal tissue. As shown in Fig. 5, for a constant oxygen background concentration, killing probability differs depending on the tissue oxygenation conditions (Vascularized or non-vascularized). In a vascularized tissue, killing probability is distributed among different parts of the tumor (and normal tissue), while in a non-vascularized tissue different layers of tumor have almost similar killing probabilities. Moreover, in a non-vascularized tissue ^1^O_2_ production is not sufficient to effectively destroy cancer cells except when tissue oxygen level becomes too high (Fig. 5b, d, f). It should be noted that in high oxygen levels (C_O2_ = 10 CO_2ref_), there is not high differences between the presence of a vascular network or not (Fig. 5e, f).

**Figure 6:**
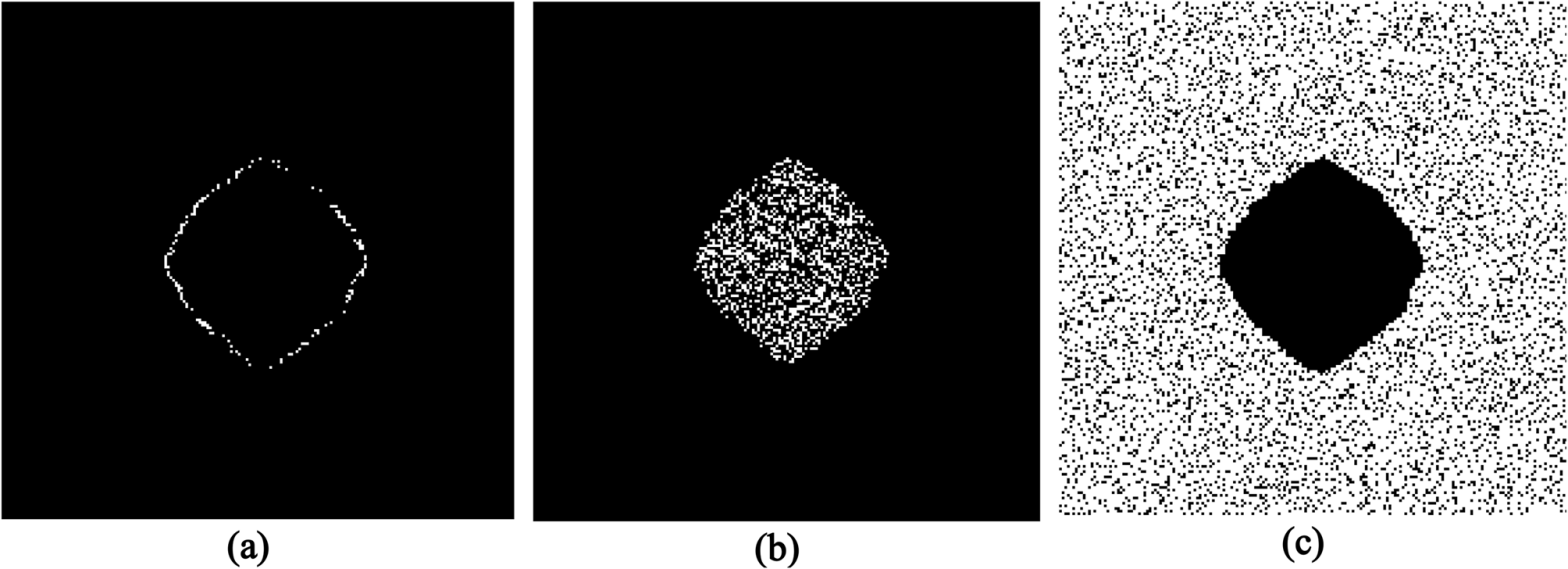
Different cells killed by RT + RS component of X-PDT includes (**a**) Proliferative and (**b**) Quiescent tumor cells as well as (**c**) Normal tissue. The white spots in (**a**) & (**b**) are tumor cells and in (**c**) are normal cells. The black spots are background area in all.

According to the results of Fig. 5, almost 50% of tumor cells will be killed by ^1^O_2_ toxicity in sufficient oxygen concentration (C_O2_ = 10 CO_2ref_) in a vascularized tumor, but this is not all X-PDT efficiency because RT component will also target the cells, simultaneously. Figure 6 shows tumor and normal cells killed by RT in the presence of 4 mg/ml nanoscintillator concentration and radiation dose 4 Gy in a normal oxygen level (CO_2ref_). According to the results, 37.26% of tumor cells killed by RT + RS that includes 3.59% proliferative cells and 33.67% quiescent cells. An interesting result is that the isolated area of tumor cells in Fig. 4(b) that ^1^O_2_ failed to destroy them, now targeted by RT + RS component of X-PDT. Killing of tumor cells in these regions emphasize on the combination effects of RT and PDT components during X-PDT.

**Figure 7:**
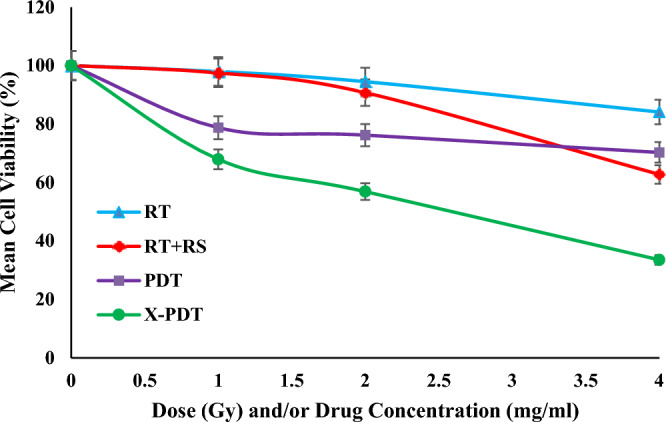
Comparison of mean tumor cell viability under different treatments of RT, RT + RS, PDT and X-PDT in a vascularized tumor with a constant tissue oxygen concentration of CO_2ref_.

Figure 7 shows the efficiency of RT, RT + RS, PDT and X-PDT under different regimes of radiation doses and/or drug concentrations in a vascularized tumor with a constant oxygen concentration of CO_2ref_. According to the results, RT alone (0–4 Gy) did not induce significant cell death, while RT in presence of radiosensitizers (RT + RS) increase cell death in drug concentrations of more than 2 mg/ml. PDT results estimate the amount of cell death happened due to ^1^O_2_ production during X-PDT and X-PDT results summarize the total effects of RT + RS, ^1^O_2_ toxicity and synergy effects of them, simultaneously. As shown, X-PDT is more effective than RT + RS and PDT alone at all doses and concentrations. As a comparison, in radiation dose of 4 Gy and drug concentration of 4 mg/ml, the cell viability dropped to 84.11% in RT only, 70.27% in PDT, 62.74% in RT + RS and 33.5 in X-PDT.

**Figure 8:**
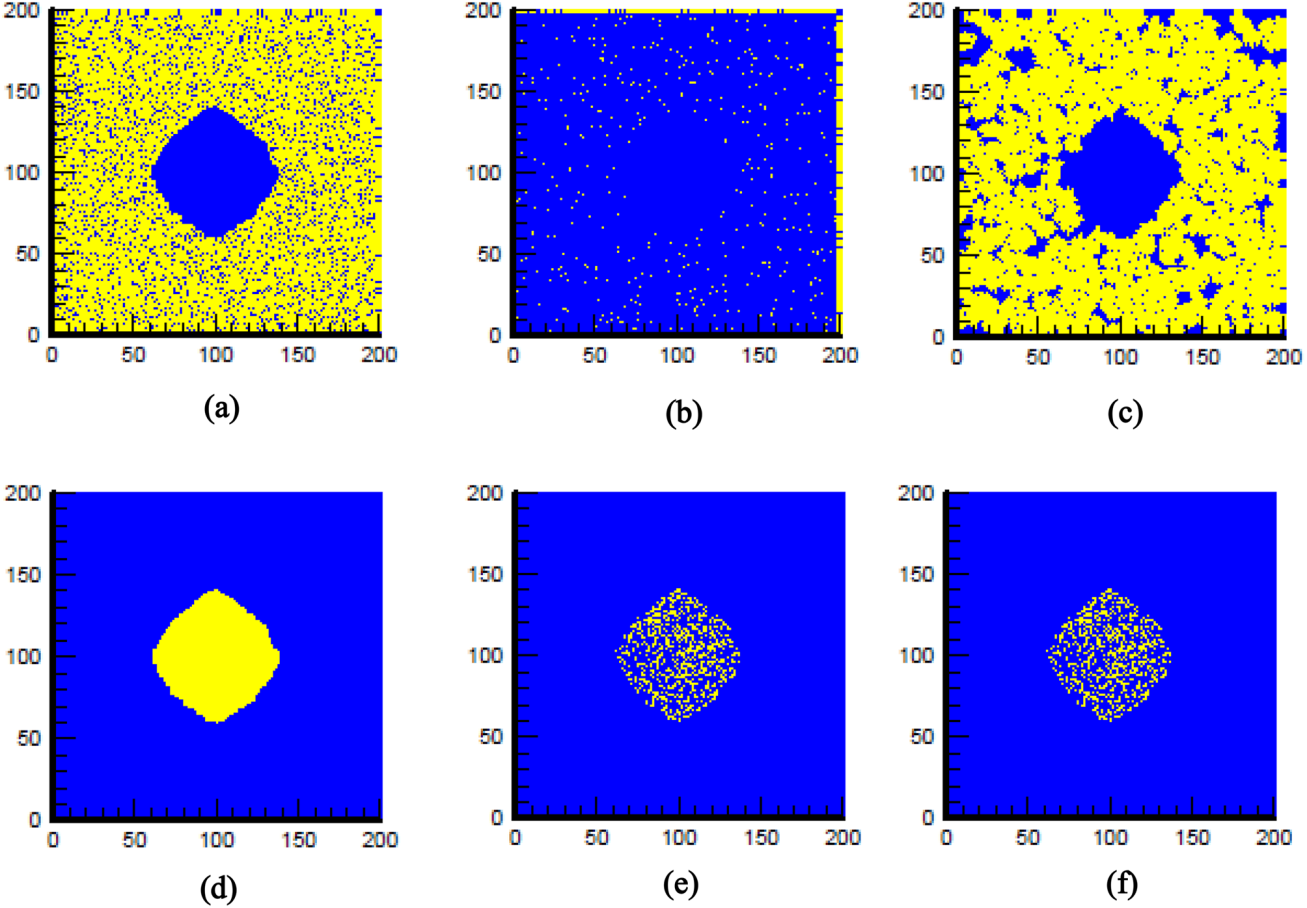
The results of X-PDT on a moderately sensitive normal tissue (**a-c**) as well as tumor cells (**d-f**) before (**a** & **d**), just after (**b** & **e**) and 4.16 days after X-PDT (**c** & **f**). The yellow color in the first row indicates normal cells and in the second row indicates tumor cells. The blue color in both rows shows the background area.

According to the Fig. 6(c), almost all of normal cells (98%) targeted by RT + RS in presence of 2 mg/ml nano-scintillator and radiation dose 4 Gy. Although most of surrounding normal cells are damaged, they are able to reconstruct themselves by cell repair mechanisms. Moreover, we assumed a strict situation that half of drug concentration remains in normal tissue under irradiation. However, treatment conditions include drug concentrations remain in healthy tissues should be optimization to reduce side effects. In pharmaceutical experiments, many efforts have been made to design ideal drug delivery systems so that drug loading in tumor cells with higher pH becomes more than normal low-pH tissues^22,23^. Figure 8 shows a moderately sensitive normal tissue and tumor cells before, just after and 4.16 days after X-PDT. Because DNA repair pathways of cancerous cells has been deregulated^24^, they are unable to repair themselves (Fig. 8) as a result, in most cases, fractionated treatments lead to complete elimination of cancerous cells. In the current situation, although some of tumor cells (35.08%) still remains after the treatment (Fig. 8e), it is possible to omit remaining cells by applying a low dose RT alternately.

**Figure 9:**
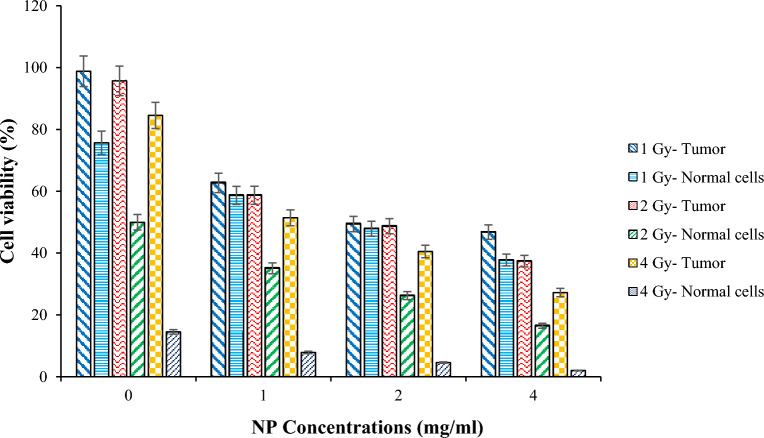
Comparison of tumor and normal cell viability under different X-PDT regimes with this assumption that half of these concentrations remains in adjacent normal tissue.

Figure 9 compares viability of tumor and normal cells under different X-PDT regimes. In comparison to the control group that receive RT only (C = 0 & D $\ne 0$), cell viability decreases as NP concentration increases. Minimum cell viability of tumor equals to about 35.08% that is related to the NP concentration of 4 mg/ml and radiation dose 4 Gy (D = 4 & C = 4). Although increasing drug concentration compared to increasing radiation dose makes more reduction in tumor cell viability, this trend is opposite in normal cells. In other words, normal cells are more sensitive to changing in radiation doses compared to changes in NP concentration. Applying dose 4 Gy in all drug concentrations decreases normal cell viability below 20%, means that it is very toxic for them while the maximum reduction for tumor cells achieved in this dose and NP concentration 4 mg/ml. Therefore, it is a decision making problem to compromise between killing tumor cells and unwanted normal tissue side effects.

For better illustration, Tables 1 and 2 present percentages of tumor and normal cells killed by ^1^O_2_ in a vascularized tissue under different oxygen concentrations and vascular network densities, respectively. Consulting the literature, we considered two reference values for tissue oxygen concentration (CO_2ref_) and vessel branching age (ψ_ref_) and compared the related results with the results in 0.25, 0.5, 2 and 4 times variations. According to the results, cell killing rate increases as tissue oxygen levels and microvascular network densities increase. Background oxygen concentration models inherent tissue differences in oxygen levels and we intended to predict why treatment efficiency differs among different cancers like colon compared to lung. Moreover, long-term effects of microvascular network densities appears in tissue oxygen levels. According to the simulations, when the tissue oxygen concentration increases to 4 CO2_ref_, 46.47% of tumor cells killed due to ^1^O_2_ toxicity that is 2 times larger than killing rate in CO2_ref_ condition. However, the rate of normal cell killing in this situation is more than 3 times compared to CO2_ref_ situation. As a result, a high tissue oxygen level although increases tumor destruction, it simultaneously affect healthy tissue destruction more effectively. Thus, optimizing treatment conditions, especially the amount of drug loading in tumor compared to healthy tissues, minimizes unwanted side effects. On the other hand, in a high density microvascular structure with ψ = 0.25 ψ_ref_ that includes about 1314 vessel segments cells killed more effective than a network with ψ = ψ_ref_ includes 53 vessel segments. Therefore, the results illustrate two important results should be considered in all ^1^O_2_ based treatments include: (1) It should be considered a tradeoff between tumor cell reduction and normal tissue side effects and (2) tissue oxygen content as well as tissue oxygenation has an important role in treatment efficiency.

**Table 1:** Percentages of different tumor cells and normal tissue killed by ^1^O_2_ during X-PDT under various tissue oxygen concentrations in dose 4 Gy and NP concentration of 4 mg/ml.

Tissue oxygen concentration (µmol/L)	Proliferative tumor cells	Quiescent tumor cells	Normal cells
CO2 = 0.65	1.03	4.94	7.71
CO2 = 2.6	0.9	6.17	8.83
CO2 = 5.2	2.54	20.19	8.39
CO2 = 10.4	3.90	34.32	11.16
CO2 = 20.8	4.41	42.06	28.42

**Table 2:** Percentages of different tumor cells and normal tissue killed by ^1^O_2_ during X-PDT under various vascular network densities in dose 4 Gy and NP concentration of 4 mg/ml.

Vessel branching age	Micro Vessel number (MVN)	Proliferative tumor cells	Quiescent tumor cells	Normal cells
**ψ** = 0.25 **ψ**_ref_	1314	3.15	32.04	12.72
**ψ** = 0.5 **ψ**_ref_	316	2.94	25.22	10.94
**ψ** = **ψ**_ref_	53	2.54	20.19	8.39
**ψ** = 2 **ψ**_ref_	21	2.47	18.27	8.80
**ψ** = 4 **ψ**_ref_	14	2.52	17.66	7.79

## Discussion

In this paper, we considered available oxygen concentration in the vicinity of X-PDT drugs to evaluate production rate of singlet oxygen in tumor microenvironment. We also estimated cell killing probability in a vascularized and non-vascularized tissue for different levels of oxygen concentrations. Combination effects of RT in targeting the isolated cells escaping from PDT, emphasized that X-PDT is more than just a PDT derivative but it is essentially a PDT and RT combination^12^. Introducing physicochemical model of X-PDT with considering tissue oxygen concentration estimated killing rates in different tumor layers (proliferative and quiescent) and illustrated why some of the cells escape from ^1^O_2_ toxicity. In this paper, we improved the existing models of X-PDT to investigate the effects of tissue oxygenation to find out the cause of different X-PDT outcomes in various tissues from the point of their oxygen content, although it did not greatly improve the number of produced ^1^O_2_. Considering variable vessel diameters, cause to produce a more realistic vascular network in providing molecular oxygen. The results showed that death rates of the cells as well as mortality patterns depend on the tissue oxygen concentration and oxygenation properties such as micro vessel density. Hope that such studies have great importance to clinical applications of X-PDT in the near futures.

## Mathematical modeling

### Overview

Development of cancer begins with a mutation in certain key genes of a normal cell. One of this mutation results is cell's runaway from normal homeostatic mechanisms causing irregular cell proliferation. After a while, over successive divisions, a cluster of about 10^6^ cancer cells forms that compete for space and nutrients. These new invading cells change the concentration of underlying molecules result in hypoxia at internal layers of tumor. Hypoxic cells secrete tumor angiogenic factors (TAFs) causing endothelial cells (EC), lining in nearby vessel walls, to form new blood vessels from the existing ones (angiogenesis)^25^. After triggering angiogenesis, blood flow penetrates into the network and provides oxygen and nutrients to the hypoxic cells. Angiogenesis process is a key feature of malignant tumors enables them to invade surrounding tissues (metastasis) and leads to challenges in their treatment. In the following, first we simulate growth of some initial tumor cells along with the consumption of chemicals include oxygen, glucose and hydrogen production to obtain appropriate concentration of molecules at the time of treatment. To model vascular tumor growth, we coupled our previous model^26^ to the nutrient diffusion model^27^ and estimate available tissue oxygen. Then, the edited model of X-PDT applied to estimate viability of cancerous cells through a feedback of tumor micro-environment. Details of each model explained in the following.

### Chemical transport

We considered the key diffusible chemicals of oxygen, glucose, hydrogen ions and VEGF. Each tumor cell is assumed to consume nutrients (glucose and oxygen) and excrete metabolic waste (hydrogen). Tumor cells also secrete VEGF under hypoxic conditions. Differential equation describes the concentration of such molecules is^28^:

1 $$\frac{\partial {C}_{i}(x,y)}{\partial t}={D}_{i}{\nabla }^{2}{C}_{i}\left(x,y\right)+{A}_{i}+ {B}_{i}$$

where *i* = *O*_*2*_*, G, H*, denotes molecules of oxygen, glucose and hydrogen ion respectively. ${D}_{i}$ are diffusion constants and functions ${A}_{i}$ and ${B}_{i}$ describe the consumption rate of molecules by the tumor cells and production rate of them by the vasculature, respectively. Also:

2 $${A}_{i}= {(\mathrm{W}}_{\mathrm{i}} \phi ) {T}_{P}$$

3 $${B}_{i}=\sum \frac{2{q}_{i}}{R} {(C}_{b,i}- {C}_{w,i}) {E}_{P,k}$$

where ${W}_{i}$ is the uptake rate of molecule *i* by cancer cells, *ϕ* denotes the density of tumor cells, ${q}_{i}$ is the vessel permeability to molecule *i*, *R* is microvessel radius, ${C}_{b,i}$ and ${C}_{w,i}$ are intravascular and wall concentrations of molecule *i*. Terms ${T}_{P}$ and ${E}_{P,k}$ are Boolean indicators represent a tumor cell locating at node *P* and a capillary segment connecting node *P* with its neighboring nodes, respectively. The summation is taken over point *P* and its adjacent nodes (*k* = *N, S, W, E*).

For the simplicity, it is assumed that the intravascular concentrations of oxygen, glucose and hydrogen ions are preserved throughout the vasculature, so ${C}_{b,i}$ has a constant value. Finally, the interstitial VEGF transport equation is defined as^27^:

4 $$\frac{\partial {C}_{v}}{\partial t}={D}_{v}{\nabla }^{2}{C}_{v}+\left({W}_{v}\upphi \right){\eta }_{P}-\left({\varepsilon }_{v}\uppsi \right){\theta }_{P}-{\lambda }_{v}{C}_{v}$$

where ${D}_{v}$ is diffusion coefficient of VEGF, ${\eta }_{P}$ and ${\theta }_{P}$ are Boolean indicators of the existence of hypoxic tumor cells and ECs on a grid point *P*, respectively, ${\varepsilon }_{v}$ is take up rate of VEGF by ECs and ${\lambda }_{v}$ is its natural decay rate.

### Cell dynamics

This model consists of three different cell types includes tumor cells, normal tissue and vasculature. The internal metabolic activities of normal and tumor cells cover glycolysis, production of acid and oxidative phosphorylation^28^. Normal cells only proliferate if density of neighbors falls below a certain value (_~_ 80%) but tumor cells can proliferate until they fill all the available spaces^29^. We adopted the previously developed models of tumor metabolism to incorporate core metabolic activities of tumor and normal cells^27,29^. Using Michaelis–Menten kinetics, oxygen consumption ${\mathrm{W}}_{\mathrm{o}}$ is determined by:

5 $${\mathrm{W}}_{\mathrm{O}2}\left({\mathrm{x,y}}\right)=-{\mathrm{V}}_{\mathrm{O}2}\frac{{\mathrm{C}}_{\mathrm{o}2}\left({\mathrm{x,y}}\right)}{{\mathrm{C}}_{\mathrm{o}2}\left({\mathrm{x,y}}\right)+{\mathrm{K}}_{\mathrm{O}2}}$$

where ${C}_{O2}$*(x,y)* is oxygen concentration,${V}_{O2}$ is maximum oxygen consumption and ${K}_{O2}$ is the half maximum oxygen concentration. Glucose consumption is also driven by:

6 $${\mathrm{W}}_{\mathrm{G}}\left({\mathrm{x,y}}\right)=-\left(\frac{{\mathrm{P}}_{\mathrm{G}}{\mathrm{A}}_{0}}{2}+\frac{27{\mathrm{W}}_{\mathrm{O}2}\left({\mathrm{x,y}}\right)}{10}\right)\frac{{\mathrm{C}}_{\mathrm{G}}\left({\mathrm{x,y}}\right)}{{\mathrm{C}}_{\mathrm{G}}\left({\mathrm{x,y}}\right)+{\mathrm{K}}_{\mathrm{G}}}$$

where ${A}_{0}$ = $\frac{29}{5}{V}_{O2}$ is the baseline production rate of ATP, and ${C}_{G}$ and ${K}_{G}$ are defined as glucose concentration, and half maximum concentration, respectively. The coefficient ${P}_{G}$ is a multiplier representing the Warburg effect (i.e. altered glucose metabolism seen in many tumor cells) where ${P}_{G}=1$ corresponds to normal glucose consumption, and ${P}_{G}>1$ denotes more glucose consumption than needed to meet normal ATP demand. Defining the target ATP production rate as $\frac{{W}_{A}}{{A}_{0}}$, where:

7 $${\mathrm{W}}_{\mathrm{A}}\left({\mathrm{x,y}}\right)=-\left(2{\mathrm{W}}_{\mathrm{G}}\left({\mathrm{x,y}}\right)+\frac{27{\mathrm{W}}_{\mathrm{O}2}\left({\mathrm{x,y}}\right)}{5}\right)$$

And

8 $${\mathrm{W}}_{\mathrm{H}}\left({\mathrm{x,y}}\right)={\mathrm{K}}_{\mathrm{H}}\left(\frac{29\left({\mathrm{P}}_{\mathrm{G}}{\mathrm{V}}_{\mathrm{O}2}+ {\mathrm{W}}_{\mathrm{O}2}\left({\mathrm{x,y}}\right)\right)}{5}\right)$$

where parameter ${K}_{H}$ accounts for proton buffering in the tumor microenvironment.

**Figure 10:**
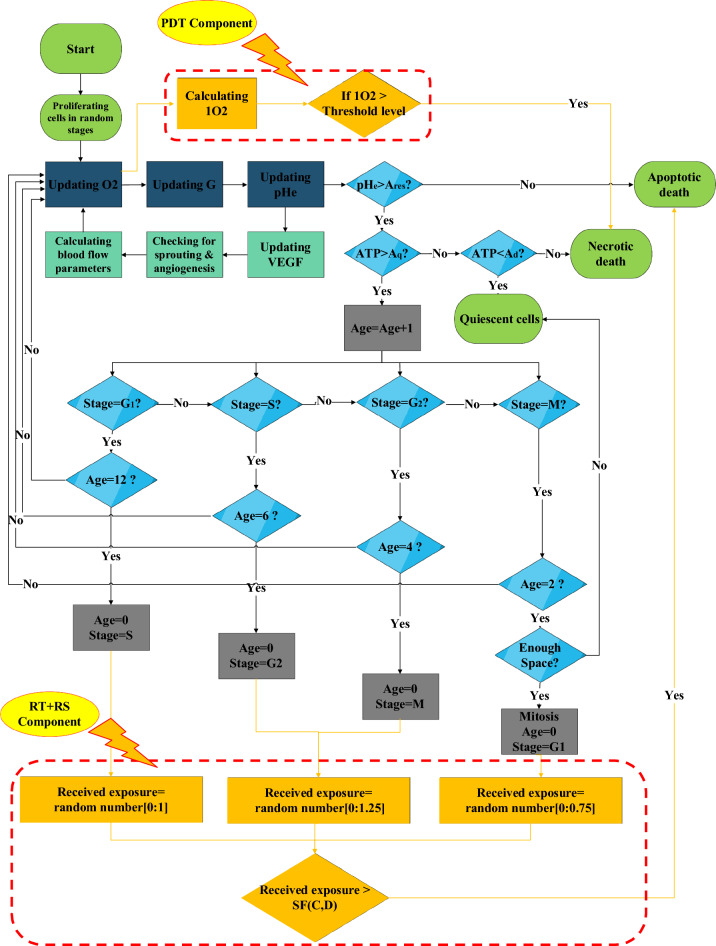
Model overview includes coupling of chemical diffusion model, cellular automaton rules and cancer cell dynamics. It is also shown how to get feedback from the model to apply the treatment at the desired time.

In the following, a set of cellular automaton (CA) rules are implemented into each cell to update its status in each time step (Fig. 10). One tumor cell per grid point is permitted either be a proliferating, quiescent, dead or treated ones. We developed some predefined rules^27^ to incorporate progression of cells in cell cycle and model the effects of radiation cell cycle sensitivity^21^. Cell cycle is consisted of four stages: Mitosis (M), Gap1 (G1), Synthesis (S), and Gap2 (G2). In each stage, the cell undergoes a different process. During Mitosis, the cell divides and produces two daughter cells. In G1 phase the cell increases in size and content. Throughout S phase DNA replication occurs and in G2 phase the cell continues to growth^30^. It is known that cells in G2 or mitosis stages are more sensitive to irradiation whereas cells in G1 stage are more resistant to radiation^31^. Figure 10 shows coupling of chemical diffusion model, cellular automaton rules, angiogenesis and blood flow model. It is also shown how to get a feedback from the tumor microenvironment to apply X-PDT components at the treatment time as will be discussed in the next sections.

### Angiogenesis

The first step of angiogenesis is nearby vessel sprouting in response to factors secreted from hypoxic tumor cells (like VEGF). These sprouts then progress in extracellular matrix (ECM). Thus, two steps can be considered in angiogenesis modeling include sprouting and progression. To model sprouting, we use our previous model of adaptive sprout spacing (ASS) to estimate the number, position and time of activated sprouts along the parent vessel^26,32^. According this model, there are two conditions for sprouts to form: 1) If the concentration of activator around each point of the parent blood vessel become greater than or equal to a chosen trigger value, and 2) the inhibitor concentration at that point be less than or equal to a chosen inhibitor threshold value. Thus, If *A* and *I* denote the angiogenesis activator and inhibitor concentrations, respectively, the required conditions for sprouting will be formulated as:

$${A}_{l}^{q}\ge {A}_{trigg,l}^{q}$$

$${I}_{l}^{q}\le {I}_{thresh,l}^{q}$$

where the subscripts specify the location on the grid and the superscripts specify the time steps.

Assuming that the activator is produced by hypoxic tumor cells, simply diffuses and decays. Inhibitor is also produced by each formed sprout and diffuses into the tissue around of it to inhibit formation of new sprouts with a random distance and then decays. Thus, the partial differential equations describing activator and inhibitor concentrations are as follows:

9 $$\frac{\partial A}{\partial t}= {D}_{a}{\nabla }^{2}A- {\lambda }_{a}A$$

10 $$\frac{\partial I}{\partial t}= {D}_{i}{\nabla }^{2}I- {\lambda }_{i}I$$

where ${\mathrm{D}}_{\mathrm{a}}$ and ${\mathrm{D}}_{\mathrm{i}}$ are activator and inhibitor diffusion coefficients, ${\uplambda }_{\mathrm{a}}$ and ${\uplambda }_{\mathrm{i}}$ are their decay rates, respectively, all taken as constants^33,34^.

Activated sprouts then progress in ECM in three ways of random motility, chemotaxis and haptotaxis^35^. Three key variables considered for progression of activated sprouts include vascular endothelial growth factor (VEGF), fibronectin and ECs. The non-dimensional equation governing the distribution of ECs is defined as^35^:

11 $$\frac{\partial \mathrm{e}}{\partial \mathrm{t}}=\nabla .\left[{D}_{e}\nabla \mathrm{e}-\mathrm{e }\left(\frac{\mathrm{x}}{1+\sigma {C}_{v}}\nabla {C}_{v}+\uprho \nabla \mathrm{f}\right)\right]$$

where *e,* ${C}_{v}$ and *f* denote non-dimensional endothelial cell density, VEGF and fibronectin concentrations, respectively. ${D}_{e}$*,* $x$ and $\rho$ are non-dimensional EC diffusion, chemotaxis, and haptotaxis coefficients, respectively. Using standard finite-difference methods, discretized form of the partial differential equations result in coefficients of the five-point finite-difference stencil to generate the probabilities of movement of an individual cell in response to its local milieu. The equation describing the interaction of sprouts with ECM fibers is:

12 $$\frac{\partial \mathrm{f}}{\partial \mathrm{t}}=\mathrm{\beta e}-\mathrm{\gamma ef}$$

where $\upbeta$ and $\upgamma$ are positive constants expressing fibronectin production and proteolytic activity of EC tip, respectively. As the activated sprouts progress in ECM, they branch and anastomosis to form a novel capillary structure. Then blood flows in the hollow capillaries to supply tumor for chemicals. Moreover, the interaction of blood with capillary network remodel their structure via wall shear stress and upregulate microvessel radius.

### Blood perfusion

Vascular network formed by angiogenesis model in previous section is a hollow network just determines the path of the endothelial cells. In order to estimate the amount of chemicals transferred by new vessels, blood flow analysis is required.

For non-Newtonian fluids, apparent viscosity μ_app_ is defined as the slope of the rheological curve at a specific shear rate. The well-known Poiseuille relation is applied to determine blood flow Q, while blood viscosity μ_app_ (R, H_D_) is defined as a function of capillary radius and hematocrit. Assuming blood flow as an incompressible flow, then mass conservation equation is applied for all nodes of the network as:

13 $$\sum_{n=1}^{k}Q=0$$

When blood flows through a flexible vessel, resistance stresses will be generated depend on the vessel radius. On the other hand, when new vessels are forming, their radius can be determined depends on the entered shear stresses as:

14 $$\Delta R={S}_{total}{R}_{old}\Delta t$$

where *Δt* is the time step. We developed the chemical transport Eq. (3) to incorporate the effect of vessel diameter on the microvessel structure and amount of oxygen transferred by the novel network. Then, according to the amount of available oxygen and in the presence of specified concentrations of drug and radiation dose, cell viability will be computed. Therefore, Eq. (14) is used to update the new vessel radius as ${R }_{new}= {R}_{old}$+$\Delta R$. The total stimulus on a considered segment corresponds to the sum of each individual stimulus, S_wss_, S_p_, and S_m_ representing the wall shear stress, intravascular pressure, and metabolic stimulus, respectively as follows^36^:

15 $${S}_{total}= {S}_{wss}+ {S}_{p}+{S}_{m}$$

We used our previous algorithm of vessel adaptation^26^ that employs an auxiliary function to predict network remodeling at specified future time steps. The updated network with new capillary radiuses, branches and flow parameters includes updated shear stresses, pressure differences and flow rates substituted with previous ones at each time step. Finally, production rates of molecules at new vascular network updated according to the new parameters.

### Treatment model

Previous studies have experimentally demonstrated that X-PDT is essentially a PDT and RT combination^12^. The two modalities target different cellular components (cell membrane by PDT and DNA by RT), leading to enhanced therapy effects. The efficacy of X-PDT is therefore due to synergy effects of RT and PDT^37^. Current models have focused only on the singlet oxygen concentration to predict treatment efficiency of X-PDT and none of them considered RT component. In the following, we model the contribution of X-PDT components and investigate the role of tissue oxygen concentration and oxygenation properties in cell viabilities.

### PDT component

**Figure 11:**
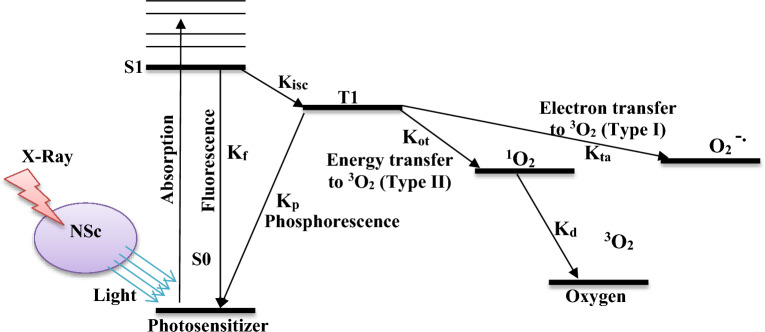
Photoactivation of photosensitizer during X-PDT by the light transmitted by nanoscintillator (NSc). ^1^O_2_ formation in type II of PDT reactions are focused for modeling PDT component of X-PDT.

Absorption of a photon by PS in its ground state, promotes it to a singlet excited state *S1* (Fig. 11). PS then lose its energy by emitting fluorescence and return to the ground state or transfer to a triplet state *T1* that has a longer lifetime. This process known as intersystem crossing (ISC) is an essential feature of a good PS. In the triplet state, PS may return to its ground state by emitting phosphorescence or transfer its energy to molecular oxygen (Type II) or transfer an electron to the biomolecules (Type I), which in subsequent reactions produces ROS^38^. Molecular oxygen that is normally in the triplet state, converted to the ^1^O_2_, during type II of PDT reactions. If the PS is not consumed in this process, the same PS molecule may create many singlet oxygen molecules. Parameter definitions and units of Fig. 11 are shown in Table 3.

**Table 3:** Parameter definitions and units of X-PDT process described in Fig. 11.

Variable	Definition	Unit
S_0_	Ground state photosensitizer concentration	mol L^−1^
S_1_	Single excited state photosensitizer concentration	mol L^−1^
T1	triplet excited state photosensitizer concentration	mol L^−1^
K_f_	Decay rate of first excited single state photosensitizer to ground state photosensitizer	s
K_p_	Decay rate of the photosensitizer triplet state	s
K_isc_	Decay rate of first excited state photosensitizer to triplet state photosensitizer	s
K_ot_	Biomolecular rate constant for reaction of ^3^O_2_ with T1	mol L^−1^ s
K_ta_	Biomolecular rate constant for reaction of T1 with biomolecules	mol L^−1^ s
K_d_	Decay rate of first excited singlet oxygen to ground state triplet oxygen	s

For modelling PDT component, we focus on the production of ^1^O_2_ as the dominant molecule during type II of PDT reactions. Because the dynamic processes of PDT are known to be very fast (_~_ μs or less)^38^, the simplified equation of ^1^O_2_ production in the time scale of a few seconds to hours, incorporates the mechanism developed by Georgakoudi et al.^39^ and the corresponding X-PDT energy transfer mechanism proposed by Klein et al.^17^ as:

16 $${C}_{1O2} = {\mathrm{N}}_{\mathrm{scint}} \left[\frac{\mathrm{photons}}{{\mathrm{cm}}^{3}}\right]\times 1\left[\frac{1\mathrm{O}2\mathrm{ molecules}}{\mathrm{photons}}\right]\times \frac{{\mathrm{C}}_{\mathrm{o}2}\left({\mathrm{x,y}}\right)}{{\mathrm{C}}_{\mathrm{o}2}\left({\mathrm{x,y}}\right)+\mathrm{ Kp}/\mathrm{Kot}}$$

Where ${\mathrm{N}}_{\mathrm{scint}}$ is density of scintillation photons emitted by a dilute suspension of nanoscintillator and C_O2_(x,y) is oxygen concentrationin at the point *(x,y)*. K_p_ is decay rate of triplet PS to the ground state and K_ot_ is the bimolecular rate of triplet PS quenching by ^3^O_2_. We assume that all photons are converted to singlet oxygen molecules. ${\mathrm{N}}_{\mathrm{scint}}$ describes energy transfer in X-PDT and depends on some physical parameters like radiation dosage d, nanoscintillator concentration ${\mathrm{C}}_{\mathrm{sc}}$, scintillator light yield ${\mathrm{Y}}_{\mathrm{sc}}$ as^17^:

17 $$\begin{aligned} {\mathrm{N}}_{\mathrm{scint}}\left[\frac{\mathrm{photons}}{{\mathrm{cm}}^{3}}\right]&=\mathrm{d}\left[\frac{\mathrm{J}}{\mathrm{kg}}\right]\times {10}^{-3}\left[\frac{\mathrm{kg}}{\mathrm{g}}\right]\times 6.2\times {10}^{12}\left[\frac{\mathrm{MeV}}{\mathrm{J}}\right]\times {\mathrm{C}}_{\mathrm{sc}}\left[\frac{\mathrm{g}}{{\mathrm{cm}}^{3}}\right]\\ &\times \frac{({\upmu /\uprho )}_{\mathrm{sc}}[\mathrm{MeV}\times {\mathrm{cm}}^{2}{\mathrm{g}}^{-1}]}{({\upmu /\uprho )}_{\mathrm{tissue}}[\mathrm{MeV}\times {\mathrm{cm}}^{2}{\mathrm{g}}^{-1}]}\times {\mathrm{Y}}_{\mathrm{sc}}\left[\frac{\mathrm{photons}}{\mathrm{MeV}}\right]\end{aligned}$$

where μ/ρ is the electron cross-sections for tissue and the nanoscintillator material can be obtained from the ESTAR database, which is maintained by the National Institute of Standards and Technologies (NIST).

Finally, the corresponding number of ^1^O_2_ production per cells (${\mathrm{N}}_{1\mathrm{O}2}$) computed by multiplying the cell volume as:

18 $${N}_{1O2}\left[\frac{1{\mathrm{O}}2 \, {\mathrm{molecules}}}{\mathrm{cells}}\right]={C}_{1O2}\left[\frac{1{\mathrm{O}}2\, {\mathrm{molecules}}} \, {{cm}^{3}}\right]\times {V}_{cell}\left[\frac{{cm}^{3}}{cell}\right]$$

Available molecular oxygen plays an important role in treatment efficiency so that quantum yield of ^1^O_2_ is affected by the amount of oxygen consumption during X-PDT^19^. The physicochemical model of X-PDT can predict treatment failure at hypoxic tumor layers. However, for any *(x,y)* of the network if *C*_*O2*_*(x,y)* >  > *K*_*p*_*/K*_*ot*_, treatment efficiency depends only on the physical parameters of Eq. (17). As well, if *C*_*O2*_*(x,y)* <  < *K*_*p*_*/K*_*ot*_, production rate of ^1^O_2_ is concerned by oxygen content of tissue, leading to unpredictable treatment effect^40,41^. Therefore, the correspondence between the oxygen content of the tissue and the selected photosensitizer can be effective in treatment efficiency. In this regards, the oxygen content of the tissue for a specific photosensitizer in different states of presence or absence of a vascular network, as well as different tissue oxygen levels, has been investigated in the results.

### Estimation of compensation coefficient

Proposed physicochemical model of X-PDT by considering oxygen concentration may not greatly improve the number of produced ^1^O_2_, but it could illustrate dependence of ^1^O_2_ yield to the available molecular oxygen. Therefore, in order to model cellular death caused by ^1^O_2_, we used invers engineering to compute the required coefficient for compensating the lack of ^1^O_2_ number from the lethal threshold value 4 × 10^7^ reported by Neider et al.^42^. We assume this compensation coefficient η_*c*_ represents all the factors that is predicted in experimental observations but have not yet been considered in modelling. Some of these factors include non-optical forms of energy transfer between nanoscintillator and conjugated photosensitizers^9^, catalyzing the radiation-induced formation of radicals and ROS by NPs^43^, enhancing radiation dose within nanometers of NP surfaces^44^. The role of all these factors applied by product a compensation coefficient η_*c*_ in Eq. (18).

### RT component

As mentioned before, each nanocomposite used in X-PDT contains a nanoscintillator and some conjugated PSs. While nanoscintillator is a light source for PS, it has also some radiation sensitizing properties^11^. In this part, we consider the effects of radiation at existence of nanoscintillators. Using nanoscintillators (radiosensitizers) can have many different mechanisms of action on effective killing of cancerous cells and improvement of treatment efficiency. Cardilin et al.^6^ proposed a model based on LQ theory and lump all of radiosensitizer processes together as having the net action of linear stimulating the radiation-induced mass transfer to make the model generally applicable. According to this model, the proportion of cancer cells surviving an irradiation dose d and plasma concentration of the radiosensitizer C equals to:

19 $$\mathrm{SF}\left(\mathrm{C},\mathrm{d}\right)={\exp}[-\left(1+\mathrm{bC}\right)\left(\mathrm{ \alpha d}+\upbeta {\mathrm{d}}^{2}\right)$$

where $\alpha$ (Gy^−1^) and $\beta$ (Gy^−2^) are cell-specific radiosensitivity parameters and *b* is a pharmacodynamic parameter associated with the radiosensitizing effects.

We call this part of X-PDT as "RT + RS component" and couple this linear stimulatory function with the tumor growth model as shown in Fig. 10 in order to estimate this component of X-PDT at the time of treatment. We also consider cell cycle sensitivity under irradiation^21^ and investigate a moderately sensitive normal tissue with $\alpha /\beta =$ 3.1^45^ to estimate treatment side effects. Moreover, we assume that normal cells have the ability to repair damages caused by radiation while the repair pathways in tumor cells is demolished due to mutation^46^.

### Initial and boundary conditions

Simulations were carried out on a 200 × 200 grid, which is a discretization of the unit square [0, 1] × [0, 1], with a space step of h = 0.005, representing a tissue with dimensions of 5 mm × 5 mm. Consulting the literature a grid size of Δx = Δy = 25 μm was set which corresponds to the approximate size of a tumor cell^47^. We initiallly assume 9 proliferative tumor cells with random stages and appropriate ages placed at the center of the domain. The rest of the lattice space is filled by normal cells with a density of 80%. A parent vessel is lining on the left border of the domain. Initial pH value of the tumor microenvironment is set to 7.4. Initial concentrations of 5.2 $\times$ 10^−6^ mol/L and 5 $\times$ 10^−3^ mol/L are set for oxygen and glucose, respectively^27^. Also, the nodal pressure difference of blood in preexisting vessels is set to ΔP = 100 mmHg with the radius of 14 µm. All capillary segments are assumed to be of initial radius and length of 2 µm and 25 µm, respectively. No flux boundary conditions are adopted for the interstitial diffusion equations. Other required parameter values are given in Table 4.

**Table 4:** Parameter values selected for mathematical modeling.

Parameter	Description	Value	Unit	References
P_G_	Upregulated glucose uptake rate	1	–	^27^
α	Probabilities of double-strand breaks in DNA of tumor cells	0.002	G^−1^	Estimated
β	Probabilities of radiation repair of tumor cells	0.01	G^−2^	Estimated
α_NC	Probabilities of double-strand breaks in DNA of normal cells	0.211	G^−1^	^48^
β_NC	Probabilities of radiation repair of normal cells	0.068	G^−2^	^48^
Y_sc_	Light yield of scintillator	10^5^	Photons/MeV	^17^
D_O2_	Tissue oxygen diffusivity	2.41 × 10^−5^	cm^2^ s^−1^	^49^
K_O2_	Half-max O_2_ concentration	5 × 10^−6^	mol L^−1^	^29^
V_O2_	Max O_2_ consumption	2.3 × 10^−16^	mol (cell s)^−1^	^50^
D_G_	Tissue glucose diffusivity	9 × 10^−5^	cm^2^ s^−1^	^51^
D_H_	Tissue hydrogen ion diffusivity	1.1 × 10^−5^	cm^2^ s^−1^	^51^
K_G_	Half-max glucose concentration	4 × 10^−5^	mol L^−1^	^29^
K_H_	Proton buffering coefficient	2.5 × 10^−4^	–	^28^
A_res_	pH Cancer cell acid resistance	6	pH	^52^
A_d_	ATP Threshold for death	0.3	–	^28^
A_q_	ATP Threshold for quiescence	0.8	–	^28^
b	Pharmacodynamic parameter associated with the radiosensitizing effect	0.45	ml/mg	^6^
C_b,o2_	Intravascular concentration of oxygen	5.6 × 10^−5^	mol L^−1^	^28^
C_b,g_	Intravascular concentration of glucose	5 × 10^−3^	mol L^−1^	^27^
C_b,H2_	Intravascular concentration of acid	3.98 × 10^−8^	mol L^−1^	^51^
ψ_ref_	Threshold of vessel branching age	18	hr	^35^
K_p_/K_ot_	The ratio of the monomolecular decay rate of the triplet state photosensitizer to the biomolecular rate of the triplet photosensitizer quenching by ^3^O_2_	11.9 × 10^−6^ (PpIx)	mol L^−1^	^39^
ϕ	Density of tumor cells	5 × 10^7^	cells cm^−3^	Calculated

## Data Availability

All data generated or analyzed during this study are included in this published article.
